# Colistin Use for the Treatment of Multi-Drug-Resistant Gram-Negative Severe Infections in ICU Patients: A Single-Center Study

**DOI:** 10.3390/jcm14030797

**Published:** 2025-01-25

**Authors:** Stanislaw Wojciech Rojek, Iga Wojtowicz, Fabio Silvio Taccone, Wieslawa Duszynska

**Affiliations:** 1 Department of Anaesthesiology and Intensive Therapy, Saint Bernard’s Hospital, Harbour Views Road, Gibraltar GX11 1AA, Gibraltar; staszek.rojek@gmail.com; 2 Department of Anaesthesiology and Intensive Therapy, University Hospital in Wroclaw, Borowska 213 Street, 50-556 Wroclaw, Poland; wojtowicz.iga@gmail.com; 3 Department of Intensive Care, Hôpital Universitaire de Bruxelles (HUB), Université Libre de Bruxelles (ULB), Route de Lennik 808, 1070 Brussels, Belgium; ftaccone@ulb.ac.be; 4 Department of Anaesthesiology and Intensive Therapy, Wroclaw Medical University, Pasteura Street 1, 50-367 Wroclaw, Poland

**Keywords:** colistin, ICU, multi-drug-resistant bacteria, infection, acute kidney injury

## Abstract

**Background:** Colistin is increasingly used to treat severe infections caused by multi-drug-resistant (MDR) bacteria, particularly in critically ill patients. Its effectiveness, especially in monotherapy, remains controversial. This study aimed to evaluate the effectiveness and toxicity of colistin therapy in severe MDR infections. **Methods:** This retrospective study included patients treated with colistin (CMS) at the ICU. Patients’ treatments were divided into four subgroups: monotherapy vs. combination therapy, empirical vs. targeted therapy, intravenous vs. intravenous plus inhaled therapy, and standard doses with and without a loading dose. The primary outcome was clinical cure. Secondary outcomes included microbiological eradication, survival rate, and drug-related toxicity, particularly acute kidney injury (AKI). Exclusion criteria included Gram-positive infection, inhaled therapy alone, use of colistin <5 days. **Results:** A total of 150 patients (mean age 60 ± 18 years, APACHE II score 17 ± 10) were included. The most frequent condition was hospital-acquired pneumonia (n = 140, 93.3%). The most common pathogen was MDR *Acinetobacter baumannii* (n = 146, 97.3%). In most patients, colistin therapy was targeted (n = 113, 75.3%) and combined with other antibiotics (n = 124, 82.7%). Inhaled CMS was added in 47 (31.3%) patients. Mean duration of therapy was 10 ± 4 days. Clinical cure occurred in 64 (42.7%) patients, microbiological eradication in 20 (13.3%). AKI developed in 65 (53.7%) patients. Inhaled CMS improved the clinical cure rates (57.4% vs. 37.0%, *p* = 0.003). **Conclusions:** Intravenous CMS was mainly used for MDR *Acinetobacter baumannii*-related *pneumonia*. Clinical cure was observed in 42.7% of patients, but renal toxicity was high. Combining intravenous and inhaled CMS may improve outcomes.

## 1. Introduction

Infections with Gram-negative multi-drug-resistant (MDR) bacteria are a growing problem for hospital management; in particular, among critically ill patients, MDR bacteria are most often responsible for ventilator-associated pneumonia (VAP) [1,2]. In this setting, some drugs are available for clinicians, at least to treat the MDR *Acinetobacter baumannii* and/or *Pseudomonas aeruginosa* strains [3]. Among those, colistin, which was first isolated in the late 1940s and therefore was almost withdrawn from clinical use because of its potential toxicity, is effective against those bacteria. The use of colistin in MDR infections, whether as monotherapy or in combination therapy, has largely been based on observational studies, leaving its effectiveness controversial [4]. Some authors argue that colistin should be used as monotherapy and only in cases where the pathogen is sensitive to the drug [3], while others report that resistance to colistin increases when it is used as monotherapy [5]. Additionally, there are findings suggesting that the most effective use of colistin is in combination with other antibiotics [6], making the optimal prescription strategy a matter of debate.

Other controversial issues regarding the use of colistin include the combination of intravenous and nebulized administration [7], as well as the use of higher than recommended daily dosages. In critically ill patients, this approach should include a loading dose (LD) of 9 million IUs, with subsequent dosing adjusted based on pharmacokinetic considerations and renal function [8]. However, combination therapy has not been associated with improved survival in patients treated with colistin for MDR *Acinetobacter baumannii* infections [6], and renal impairment has been observed in up to 60% of patients, particularly with higher doses [9].

Given these issues, there is a growing need for additional data on the practical use of colistin in the treatment of MDR infections, particularly in terms of its effectiveness and potential toxicity. Therefore, the aim of this study was to evaluate the use of colistin in the treatment of critically ill patients with multi-resistant bacterial infections, focusing on clinical and microbiological responses, dosing effects, and associated side effects.

## 2. Methods

### 2.1. Study Population

This retrospective study included consecutive patients treated with colistin at the Department of Anaesthesiology and Intensive Therapy of the University Hospital in Wroclaw, Poland, between January 2014 and December 2019. The study was carried out as part of a project approved by the Bioethics Committee of the Medical University of Wroclaw (Protocol Number 580/2016 and 655/2018). Patients’ written consent was waived because of the retrospective design of the study and the anonymized data collection.

Inclusion criteria were as follows: (a) prescription of colistin (Colistin TZF, Tarchomińskie Zakłady Farmaceutyczne “Polfa”, Warsaw, Poland), given intravenously, alone or in combination with inhaled administration; (b) infections related to Gram-negative MDR strains. Exclusion criteria included the following: (a) Gram-positive bacterial infection; (b) inhaled therapy alone; (c) use of colistin for less than 5 days.

### 2.2. Data Collection

Based on the analysis of the medical history and electronic documentation of patients, information was collected on the following: demographics; reason for ICU admission; the severity of the patient’s condition, according to the APACHE (Acute Physiology and Chronic Health Evaluation) II score [10]; type of infection; antibiotic therapy (i.e., targeted vs. empirical therapy—monotherapy vs. combination therapy with another drug); type of pathogen; initiation and duration of colistin treatment; cumulative colistin dose. The decision to administer colistin was taken after a multi-disciplinary discussion including the ICU attending physician, a microbiologist, and a specialist in infectious diseases. The following biochemical and clinical parameters (i.e., white blood cells count; C-Reactive Protein, CRP; procalcitonin, PCT; serum creatinine; body temperature) measured on the day of colistin initiation and all the consecutive days until the end of treatment were also collected.

### 2.3. Infection Diagnosis

Nosocomial infections were diagnosed according to ECDC (European Center Disease Control, Solna, Sweden) criteria in patients hospitalized >48 h [11]. Ventilator-associated pneumonia (VAP), non-ventilation hospital-acquired pneumonia (NV-HAP) and urinary tract infection (UTI), central nervous system (CNS), central line-associated bloodstream infection (CLA-BSI), intra-abdominal infection (IAI), and sepsis was defined on a basis of international, clinical and microbiological criteria [8,11,12]. Two doctors from the ICU and one microbiologist participated in the infection diagnosis. Qualitative and quantitative microbiological diagnostics of bronchial secretions (collected using the mini-broncho-alveolar lavage method) as well as blood, urine, and other body fluids were carried out in compliance with Good Laboratory Practice principles and EUCAST (European Committee on Antimicrobial Susceptibility Testing) standards of microbiological diagnostics, at the UH Microbiological Laboratory [13]. The sensitivity of bacterial strains to colistin with the determination of the MIC (Minimal Inhibitory Concentration) value was carried out according to the EUCAST criteria using the dilution method [13,14]. In the microbiological diagnostics of infections, rapid methods of pathogen identification (FILMARRAY respiratory, blood, or cerebral panels, BioFire Diagnostics, Salt Lake City, UT, USA, and MaldiTOF Biotyper, Bruker, Billerica, MA, USA) were also used [14]. An MDR pathogen was defined as a bacterium that has acquired resistance to at least one drug in three or more antimicrobial classes [15].

### 2.4. Study Outcomes

The primary outcome of this study was the occurrence of clinical cure. Secondary outcomes included microbiological eradication, survival at ICU discharge, and lack of reinfection within 14 days after the end of colistin treatment. Also, data on the occurrence of acute kidney injury (AKI) [16] and the initiation of continuous renal replacement therapy (CRRT) were collected.

Clinical cure was defined as the complete resolution of the infection, along with its associated signs and symptoms, without recurrence, clinical deterioration, or the need for additional antibiotic therapy. The diagnosis of infection and the assessment of clinical cure were made collaboratively by two ICU physicians and a microbiologist. Clinical cure was established on the basis of the improvement in the patient’s clinical conditions, i.e., improvement in organ dysfunction using the Sequential Organ Failure Assessment Score (SOFA) associated with the normalization of X-rays, improvement in oxygenation and auscultation normalization over the lung fields; improvement in abdominal symptoms; absence of significant bacterial growth in urine sample (<10^2^/mL colonies) or/and cerebrospinal fluid (less than 5/mL of white blood cells and the absence of pleocytosis and bacterial strains); normalization of inflammatory markers after treatment completion (white blood cells < 12,000/mm^3^; CRP < 7 mg/L; PCT < 0.5 ng/mL; body temperature < 37 °C). Microbiological eradication was defined as the absence of growth of the causative pathogens in cultures from the infection site, one day after completing therapy. Reinfection was identified if the same pathogen was involved in another infectious process within 14 days after clinical recovery, requiring the administration of antibiotics.

### 2.5. Statistical Assessment

Statistical analyses were performed using IBM SPSS Statistics 25.0. The chi-square test of independence or Fisher’s exact test was used for categorical variables, with a significance level set at 5%. Descriptive statistics and frequency analyses were employed to characterize the study population. For quantitative variables, the mean and standard deviation or median and interquartile range were reported. For qualitative variables, frequency distributions were described using counts and percentages. Analyses were conducted for the entire sample and stratified into four subgroups: (a) monotherapy vs. combination therapy, (b) empirical vs. targeted therapy, (c) intravenous vs. intravenous plus inhaled therapy, and (d) high dose daily regimen with a loading dose (LD) vs. standard doses without an LD. Acute kidney injury (AKI) occurrence was also analyzed in a subset of patients not on continuous renal replacement therapy CRRT and/or hemodialysis on the first day of colistin treatment, according to the baseline creatinine clearance calculated using the Cockcroft–Gault formula (CrCL; <50 mL/min vs. ≥50 mL/min) [17].

## 3. Results

### 3.1. Study Population

Over the study period, 150 patients (n = 45 female, 30.0%), with a mean APACHE II score on admission of 17, were included. The characteristics of the study population are presented in Table 1. One hundred seventy-nine infections were identified in the study group, with 29 (19.3%) patients with more than one infection. The most frequent pathogen was *Acinetobacter baumannii* (n = 146, 97.3%). Colistin was most often used to treat NV-HAP or VAP, which occurred in 140 (93.3%) patients (n = 60 and n = 80, respectively). One third of patients were in septic shock.

### 3.2. Colistin Therapy

The mean duration of colistin therapy was 10 ± 4 days. Targeted therapy was given in 113 (75.3%) patients, while empirical was given only in 37 (24.7%). Combination therapy with other antimicrobial agents was administered in 124 (82.7%) patients, monotherapy in 26 (17.3%). The most common drug given in combination with colistin were meropenem (n = 22) or imipenem (n = 66), amikacin or gentamicin (n = 36), cefoperazone/sulbactam (n = 7), and ampicillin/sulbactam (n = 4). While the isolated strains were all susceptible to colistin, additional antibiotics were considered as effective in vitro only in 38 out of 124 patients (30.6%). Inhaled colistin was used in combination with intravenous therapy in 47 (39.2%) patients. A higher daily regimen including an LD (e.g., 9 million IU) was used in 20 (13.3%) patients.

### 3.3. Primary and Secondary Outcomes

Clinical cure was observed in 64 (42.7%) of patients. Microbiological eradication was observed in 20 (13.3%), survival at ICU discharge in 97 (64.7%), and lack of reinfection in 72 (48.0%) patients. Persistent microbiological colonization occurred in seventy-seven (51.3%) patients and only one patient developed resistance to colistin during treatment.

On the initiation of colistin therapy, 29 (19.3%) patients were already on CRRT or hemodialysis; among the 121 remaining, 27 (22.2%) had a CrCl < 50 mL/min. A total of 65/121 (53.7%) patients presented AKI after the initiation of colistin therapy; in particular, AKI occurred in 21/27 patients (77.8%, all requiring CRRT) with baseline CrCL < 50 mL/min and in 44/94 (46.8%, with 23 requiring CRRT) patients with baseline CrCL > 50 mL/min (*p* = 0.04). The median time of AKI occurrence from the initiation of colistin therapy was 11 (IQR 14-8) and 10 (IQR 14-7) days in the two CrCL groups, respectively. The recovery of renal function during the ICU stay was found in 14 out of 65 (21.5%) AKI patients.

### 3.4. Subgroup Analyses

**Figure 1:**
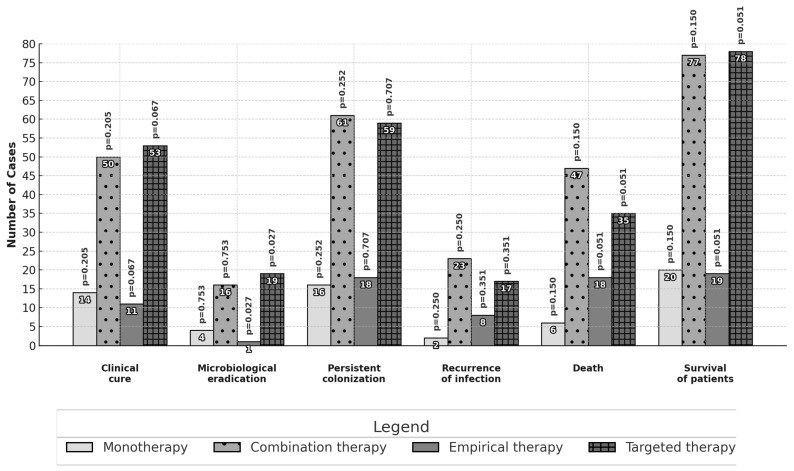
Comparison of outcomes: colistin in monotherapy or combination therapy and empirical or targeted therapy. The analysis was performed with the chi-square test or Fisher’s exact test.

**Figure 2:**
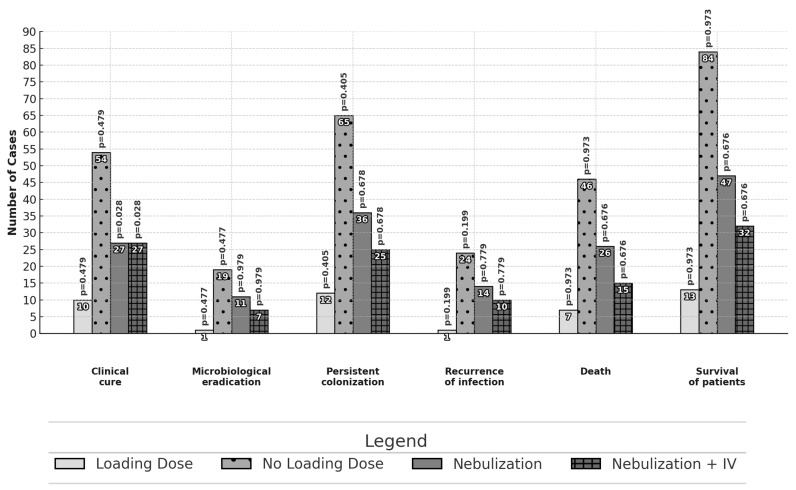
Comparison of outcomes: use or non-use of the colistin loading dose and route of administration—intravenously or nebulization with intravenous infusion. The analysis was performed with the chi-square test or Fisher’s exact test.

Differences in the primary and secondary outcomes between different subgroups are reported in Figure 1 and Figure 2. No statistically significant differences in effectiveness were observed among patients treated with monotherapy or combination therapy as well as among patients treated with high or standard dose daily regimens. Patients receiving a targeted therapy presented a higher microbiological eradication than those receiving empirical therapy (16.8% vs. 2.7%, *p* = 0.027). Patients treated with inhaled and intravenous therapy had a higher proportion of clinical cure outcomes than those with intravenous therapy alone (57.4% vs. 37.0%; *p* = 0.028).

## 4. Discussion

This study assessed the use of colistin in the ICU setting to treat mainly MDR *Acinetobacter baumannii* pneumonia. We observed clinical cure in nearly 50% of patients, with an observed increased clinical cure with the combination of inhaled and intravenous administration. The occurrence of AKI was high, with a small proportion of patients showing recovery of renal function over the ICU stay.

In this study, similarities were observed with an Italian multi-center study regarding the use of colistin, which was administered as targeted therapy in 76% of patients, and in combination with other antibiotics in 80% of cases [18]. The high use of colistin in targeted therapy may reflect hospital and departmental protocols recommending its use for severe systemic infections caused by susceptible Gram-negative bacteria. In the present analysis, the most common combination therapy involved colistin and a carbapenem. Literature data suggest that combining colistin with rifampicin or with both rifampicin and carbapenem enhances clinical effectiveness in treating MDR *Acinetobacter baumannii* VAP, with reported success rates between 76 and 100%. Although combinations of colistin with tigecycline or rifampicin were not observed in this study, likely due to the unavailability of intravenous rifampicin and tigecycline’s lack of approval for pulmonary infections in Poland, colistin was frequently combined with sulbactam; this reflects evidence supporting the efficacy of high-dose sulbactam (doses > 6 g) in treating MDR *Acinetobacter baumannii* infections [3].

The effectiveness of colistin treatment in this study was evaluated using several clinical parameters, including clinical cure, microbial eradication, recurrence of infection, and mortality. The low percentage of microbial eradication may suggest persistent colonization with the pathogen despite treatment. These findings are consistent with, though somewhat different from, those of Kofteridis et al., where clinical cure was observed in 32.5% of patients, microbial eradication in 50%, recurrence of infection in 6%, and mortality in 42% of patients treated with intravenous colistin for VAP [19]. The notable discrepancy in microbial eradication (13.3% vs. 50%) may highlight differences in study populations or treatment protocols. In studies conducted in Greece and Spain, the use of colistin (3 million IU every 8 h without a loading dose) for *Pseudomonas aeruginosa* and *Acinetobacter baumannii* infections showed clinical cure rates of 73%, 74.4%, and 79%, respectively [3]. Similarly, a higher clinical response rate (73%) was reported in a cohort of 28 patients with sepsis caused by similar pathogens when treated with the same colistin regimen, though 42.3% of patients ultimately died, slightly more than in our study [20]. In another study from Italy, colistin was used with a loading dose of 9 million IU and a maintenance dose of 4.5 million IU every 12 h, resulting in an 82.1% clinical cure rate [21]. These findings suggest that higher doses and the inclusion of a loading dose may improve clinical outcomes, although further research is needed to optimize treatment strategies for critically ill patients.

The evaluation of colistin use in empirical versus targeted therapy in this study did not reveal statistically significant therapeutic advantages for empirical treatment. However, patients who received targeted therapy showed numerically higher clinical cure rates along with a lower recurrence of infection and lower mortality rates. Previous studies comparing colistin in empirical versus targeted therapy, often with carbapenems, have also yielded mixed results [22,23]. Based on current literature, empirical colistin therapy may be beneficial in life-threatening cases, especially in settings with a high prevalence of MDR strains, which seems theoretically justified [3]. This study also found no significant advantage in using colistin in combination with other antibiotics compared to colistin monotherapy. Monotherapy was even associated with numerically higher survival rates, higher clinical cure rates, and a lower recurrence of infection. The literature on this topic is diverse and often contradictory. For instance, Falagas et al. found no difference in clinical response between colistin monotherapy and combination therapy with meropenem, though monotherapy was associated with significantly lower mortality (0% vs. 36.8%, *p* = 0.007) [24]. Conversely, Qureshi et al. reported improved outcomes with combination therapy (57.1% vs. 14.3%) [25]. A multi-center study in Turkey also demonstrated better survival and microbial eradication rates with combination therapy [26]. However, a randomized international trial comparing colistin monotherapy with combination therapy for pneumonia and bacteremia caused by *Acinetobacter baumannii* did not show any significant difference in clinical failure between the two approaches [27].

Regarding the use of a loading dose (LD) of colistin, this study did not find a significant effect on mortality or clinical cure rates, though it may have contributed to a reduction in infection recurrence, observed in only 5% of patients who received an LD. The literature remains inconclusive on this topic. Dalfino et al. reported much higher clinical cure rates with a colistin LD regimen compared to our study, with an even larger discrepancy in microbial eradication [21]. However, *Acinetobacter baumannii* infections accounted for only 46.4% of cases in that study, compared to 97.3% MDR strains in our study population. Conversely, a prospective cohort study of 255 patients with *Acinetobacter baumannii* MDR infection found no significant difference in clinical cure rates between those receiving an LD and those without an LD [28]. The high rate of persistent colonization observed in our cohort warrants further investigation.

Nebulization of colistin achieves much higher drug concentrations at the infection site, and studies like those by Lu et al. suggest that VAP caused by *A. baumannii* MDR and *P. aeruginosa* MDR strains can be effectively treated with nebulized colistin at doses of 5 million IU every 8 h [29]. We observed a higher clinical cure with such combined therapy, although no significant differences regarding survival, microbial eradication, recurrence of infection, or persistent colonization were observed. Similar studies, such as one conducted in Greece, found no significant benefits from adding nebulized colistin to IV therapy for microbial eradication, mortality, or clinical cure [19]. Another study in Turkey also reported no differences in clinical cure, recurrence, microbial eradication, or mortality between the two forms of therapy [30]. While our findings regarding microbial eradication and mortality are consistent with these studies, our results showed a higher clinical cure rate with combined therapy.

The impact of colistin on renal function was also examined. In this study, 53.7% of patients experienced a deterioration in renal function, a result consistent with other clinical trials, where AKI rates ranged from 5% to 60% [3,9]. Only 21% achieved full recovery of renal function and the deterioration in renal function was more common in patients with baseline creatinine clearance <50 mL/min. The relationship between colistin therapy duration and nephrotoxicity is complex. Some studies suggested early kidney damage during colistin treatment, while others highlighted the cumulative dose as a risk factor. However, other research shows no clear link between the duration of colistin therapy and renal impairment [3].

According to the latest guidelines and available literature [31,32,33,34,35], colistin continues to play a crucial role in the treatment of infections caused by Gram-negative MDR pathogens, despite the introduction of new antibiotics such as the following: ceftazidime–avibactam, meropenem–vaborbactam, cefiderocol, aztreonam–avibactam, eravacycline.

This study has several limitations that should be acknowledged. First, being a single-center study, the pathogens responsible for nosocomial infections, as well as their sensitivity to colistin, may differ from those in other settings. This variability could influence clinical outcomes such as cure rates, survival, and microbial eradication. Second, the study primarily focused on respiratory tract infections, and the efficacy of colistin in other clinical forms of infection may vary. Third, caution is needed when interpreting survival outcomes, as multiple factors influence mortality in ICU patients, beyond the use of colistin alone. Fourth, the limited number of patients receiving a loading dose prevented an in-depth evaluation of its impact, particularly in relation to the cumulative dose and the development of AKI. Although the loading dose has been associated with a risk of renal impairment in other studies [3,9], this analysis was not possible in the present study. Fifth, we primarily treated *Acinetobacter*-related infections, and no additional conclusions can be drawn for other pathogens. Sixth, the number of patients in the compared groups differed; however, the groups were homogenous with respect to other specific criteria. Statistical analysis was feasible despite the differences in group sizes, but the results should be interpreted with caution. Lastly, the study did not assess the specific effects of colistin when combined with individual antibiotics on clinical, microbiological, or survival outcomes. This remains an important area for future investigation, as combination therapy with colistin continues to be of clinical interest.

## 5. Conclusions

In this single-center study, colistin was primarily used to treat pneumonia caused by MDR *A. baumannii*, often in targeted and combined therapy, with clinical cure rates of 43%. Combined/empirical therapy offered no significant advantage over monotherapy/targeted therapy. Colistin use with a loading dose reduced infection recurrence, and its combination of inhaled and intravenous administration resulted in higher clinical cure rates, though renal function deterioration occurred in 50% of patients, especially within 10 days of therapy.

## Figures and Tables

**Table 1:** Characteristics of the study group according to different subgroups. Data are presented as count (%), mean (SD), or median (IQRs).

Therapy				Full Test *n* = 150	Variables
Targeted *n* = 113	Empirical *n* = 37	Combination *n* = 124	Monotherapy *n* = 26		
					**Sex, *n* (*%*)**
31 (27.4)	14 (37.8)	37 (29.8)	8 (30.8)	45 (30.0)	Women
82 (72.6)	23 (62.2)	87 (70.2)	18 (69.2)	105 (70.0)	Men
59.1 (18.7)	63.7 (16.1)	60.3 (18.5)	60.0 (16.3)	60.3 (18.1)	Age, *M* (*SD*)
18.0 (13.0–23.0)	17.0 (13.5–23.0)	17.5 (13.0–23.0)	17.0 (13.5–23.0)	17.0 (13.0–23.0)	APACHE II, *Me* (*IQR*)
78 (69.0)	19 (51.4)	77 (62.1)	20 (76.9)	97 (64.7)	Survival, *n* (*%*)
29.0 (17.0–41.0)	24.0 (16.0–36.0)	28.0 (17.0–41.0)	28.0 (15.0–43.5)	28.0 (17.0–40.5)	Time of ICU stay, Me (IQR)
33.8 (29.6)	25.5 (15.1)	30.4 (17.5)	38.3 (52.8)	31.8 (27.0)	Time of ICU stay, *M* (*SD*)
69 (61.1)	23 (62.2)	77 (62.1)	15 (57.7)	92 (61.3)	Medical patients, *n* (%)
44 (38.9)	14 (37.8)	47 (37.9)	11 (42.3)	58 (38.7)	Surgical patients, *n* (%)
32 (28.3)	15 (40.5)	42 (33.9)	5 (19.2)	47 (31.3)	Sepsis/Septic shock, *n* (%)
112 (99.1)	36 (97.3)	124 (100.0)	24 (92.3)	148 (98.7)	Mechanical ventilation, *n* (%)
70 (61.9)	28 (75.7)	85 (68.5)	13 (50.0)	98 (65.3)	Circulatory failure, need for the use of catecholamines, *n* (%)
14 (12.4)	3 (8.1)	0 (0)	17 (13.7)	17 (11.3)	Other nephrotoxic agents, *n* (%)
28 (24.8)	8 (21.6)	35 (28.2)	1 (3.8)	36 (24.0)	Aminoglycosides, *n* (%)
62 (54.9)	18 (48.6)	73 (58.9)	7 (26.9)	80 (53.3)	Vancomycin, *n* (%)
42 (37.2)	21 (56.8)	54 (43.5)	9 (34.6)	63 (42.0)	Diuretics, *n* (*%*)
n = 141	n = 38	n = 149	n = 30	n = 179	The type of infection, *n* (%)
45 (31.9)	15 (39.5)	51 (34.3)	9 (30.0)	60 (33.5)	HAP
62 (44.0)	18 (47.5)	67 (45.0)	13 (43.3)	80 (44.7)	VAP
6 (4.3)	1 (2.6)	7 (4.7)	0 (0)	7 (3.9)	CLA-BSI
5 (3.5)	1 (2.6)	6 (4.0)	0 (0)	6 (3.4)	Peritonitis
7 (5.0)	1 (2.6)	3 (2.0)	5 (16.7)	8 (4.5)	UTI or urosepsis
2 (1.4)	1 (2.6)	3 (2.0)	0 (0)	3 (1.6)	OUN Infection
14 (9.9)	1 (2.6)	12 (8.0)	3 (10.0)	15 (8.4)	Others
					Pathogen detected, *n* (%)
112 (99.1)	34 (91.9)	120 (96.8)	26 (100.0)	146 (97.3)	*Acinetobacter baumannii*
1 (0.9)	3 (8.1)	4 (3.2)	0 (0)	4 (2.7)	*Pseudomonas aeruginosa*
37.1 (0.8)	36.8 (0.6)	37.0 (0.8)	36.8 (0.7)	37.0 (0.8)	Temperature C, *M* (*SD*)
12.7 (8.2–19.1)	15.1 (9.8–30.9)	14.1 (9.5–21.9)	11.0 (6.3–16.1)	13.3 (8.9–20.4)	WBC 10^3^/mm^3^, *Me* (*IQR*)
1.3 (0.4–5.1)	4.6 (0.7–23.1)	1.9 (0.5–8.2)	0.6 (0.1–5.1)	1.6 (0.5–7.7)	PCT ng/mL, *Me* (*IQR*)
161.4 (102.6–238.9)	141.3 (109.0–266.9)	174.0 (106.5–250.7)	122.9 (59.6–211.8)	161.4 (102.6–243.0)	CRP ng/mL, *Me* (*IQR*)
0.8 (0.6–1.5)	1.3 (0.8–2.2)	1.0 (0.7–1.8)	0.8 (0.6–1.1)	0.9 (0.7–1.8)	Serum creatinine at the initiation of the treatment mg/dL, *Me* (*IQR*)
85.5 (54.9–113.7)	49.0 (28.0–140.9)	78.5 (35.1–131.7)	98.0 (68.7–133.4)	81.0 (38.0–131.3)	Creatinine clearance at the initiation of the treatment, mL/min, *Me* (*IQR*)

CLA-BSI: central line-associated bloodstream infection; VAP: ventilator-associated pneumonia; UTI: urinary tract infection; n: number of patients: *M*: average value; *Me*: median; *IQR*: interquartile range; *SD*: standard deviation.

## Data Availability

Data is unavailable due to privacy and legal restrictions.

## Abbreviations

ATS: American Thoracic Society; AKI: acute kidney injury; AKIN: acute kidney injury network; APACHE: acute physiology and health evaluation; BAL: broncho-alveolar lavage; BT: body temperature; CDC: Centers for Disease Control; CMS: colistin metaneosulfate; CRRT: continuous renal replacement therapy; CRP: C-reactive protein; CrCl: creatinine clearance; EUCAST: European Committee on Antimicrobial Susceptibility Testing; IDSA: Infectious Diseases Society of America; ICU: intensive care unit; HAP: hospital-acquired pneumonia; LD: loading dose; MBL-MD: maintenance dose; MDR: multi-drug-resistant; MIC: minimum inhibitory concentration; NHSN: National Healthcare Safety Network; PD: pharmacodynamics; PK: pharmacokinetics; PCT: procalcitonine; VAP: ventilator-associated pneumonia; WBC: white blood cells.
